# Reach of Community-Selected Strategies to Reduce Opioid-Related Overdose Deaths in the HEALing Communities Study

**DOI:** 10.1080/10826084.2025.2549496

**Published:** 2025-09-02

**Authors:** Megan E. Hall, LaShawn Glasgow, JaNae Holloway, Rouba A. Chahine, Jill Davis, Miriam T. H. Harris, Hannah K. Knudsen, Jessica L. Neufeld, Emmanuel Oga, David W. Lounsbury, Nasim Sabounchi, Alissa Davis, Marissa Smith, Rachel P. Chase, Sylvia A. Ellison, Judy Harness, Hilary L. Surratt, Sharon L. Walsh, Peace Julie Nakayima, Redonna Chandler

**Affiliations:** a RTI International, Research Triangle Park, North Carolina, USA; b The Ohio State University, Columbus, Ohio, USA; c Boston University Chobanian & Avedisian School of Medicine, Boston, Massachusetts, USA; d University of Kentucky, Lexington, Kentucky, USA; e Columbia University, New York, New York, USA; f Albert Einstein College of Medicine, Bronx, New York, USA; g CUNY Graduate School of Public Health and Health Policy, New York, New York, USA; h National Institute on Drug Abuse, National Institutes of Health, Bethesda, Maryland, USA

**Keywords:** Opioids, overdose, community intervention, implementation science, reach

## Abstract

**Introduction:**

Despite recent declines, the U.S. opioid overdose crisis persists. The HEALing Communities Study (HCS) aimed to reduce opioid overdose deaths through community-level adoption of evidence-based practices (EBPs), including overdose education and naloxone distribution (OEND) and medications for opioid use disorder (MOUD). This paper describes the reach of OEND and MOUD strategies implemented by the 33 HCS wait-list control communities.

**Methods:**

We conducted descriptive statistical analysis of reach data collected from July 2022 through December 2023 to (1) summarize overall EBP implementation and reach and (2) compare the demographic representation of individuals reached to community demographic population estimates.

**Results:**

Communities implemented 474 EBPs (251 OEND, 223 MOUD), reporting an average reach of 16,482 individuals monthly. For OEND, percent reached exceeded community population representation for individuals aged 18–34 years (40% vs 31%) and 35–54 years (41% vs 31%) and non-Hispanic Black individuals (20% vs 15%). For MOUD, percent reached exceeded the opioid use disorder population representation for individuals aged 35–54 years (58% vs 52%) and non-Hispanic White individuals (88% vs 80%); however, there was lower representation for individuals aged 18–34 years (28% vs 38%) and non-Hispanic Black individuals (6% vs 13%). Distributions by sex were comparable for OEND and MOUD.

**Conclusions:**

Findings signal the promise of community-engaged interventions to reach diverse groups with OEND and reflect the challenges of overcoming longstanding barriers to MOUD access. This work provides practical examples for monitoring the reach of EBPs across multiple research sites and communities and for assessing representativeness, a valuable marker of equity.

**Trial Registration:**

Clinical Trials.gov http://www.clinicaltrials.gov: Identifier: NCT04111939

## Introduction

Despite recent declines, opioid-related overdose deaths (OODs) have been a persistent public health problem in the United States (Ahmad et al., 2025). From 1999 to 2022, almost 730,000 people died from an overdose involving prescription or illegal opioids (Centers for Disease Control and Prevention, 2025). Over the course of overlapping waves of OOD, the crisis disproportionately impacted certain geographic areas and groups that have been historically marginalized by the health care system, including Black, Hispanic, and Native American populations (Friedman & Hansen, 2022; Han et al., 2022; Hoopsick et al., 2021; Post et al., 2022). Addressing the opioid overdose burden requires increasing the adoption of evidence-based practices (EBPs) for preventing OODs, including overdose education and naloxone distribution (OEND) (Clark et al., 2014; Giglio et al., 2015) and medication treatment for opioid use disorder (MOUD) (Babu et al., 2019; Larochelle et al., 2018; Mattick et al., 2014; Sordo et al., 2017).

Unfortunately, a wide range of factors, including organizational barriers, workforce issues, structural racism, stigma, and complex regulations, impede equitable access to life-saving OEND and MOUD interventions (Calcaterra et al., 2019; Goedel et al., 2020; Haffajee et al., 2018; Livingston et al., 2018; Netherland & Hansen, 2017; Suen et al., 2024; Zang et al., 2023). For example, in the United States, methadone, one of the three medications approved by the Food and Drug Administration (FDA) for the treatment of opioid use disorder, can only be dispensed by an accredited and certified opioid treatment program (SAMHSA, 2025). The approximately 2,000 opioid treatment programs across the country are too few to meet the needs of the millions of individuals with opioid use disorder who could benefit from methadone treatment (Calcaterra et al.; Dowell et al., 2024; Federal Register, 2024). The full accreditation and certification process for opioid treatment programs can take a year or more, and some states impose licensing requirements and regulations that are more inhibitory than federal policies (SAMHSA 2025; Suen et al. 2024). The highly regulated process of dispensing methadone through opioid treatment programs poses challenges for establishing more programs to enhance access to methadone (Calcaterra et al.). In some cases, local or organizational policies impose additional restrictions related to the locations and operating hours, which can also limit access to treatment (Suen et al. 2024).

Furthermore, patient-level barriers, including lack of insurance, housing, transportation, and other health-related social needs limit utilization of OEND and MOUD services (Peet et al., 2022; Pullen & Oser, 2014). Expanding the methadone example, people living in rural areas may need to travel long distances daily to reach an opioid treatment program, a barrier that is exacerbated in cases where transportation options are limited (Calcaterra et al., 2019; Suen et al., 2024). While there are some take-home flexibilities for methadone that can help address transportation-related barriers, this option also has restrictions and is not available in every state (Federal Register, 2024). Unlike methadone, naltrexone and buprenorphine—the other FDA-approved medications for the treatment of opioid use disorder—can be dispensed by pharmacies and office-based practices. However, providers of naltrexone and buprenorphine are predominately located in neighborhoods where the majority of residents have middle and higher incomes and are white (Goedel et al., 2020; Guerrero et al., 2022). Additionally, it is estimated that public insurance is accepted by only about half of physicians who can prescribe naltrexone and buprenorphine (Guerrero et al.; Knudsen & Studts, 2019). Ultimately, the complex interplay of multi-level factors results in suboptimal reach for MOUD. In 2022, among adults 18 years of age and older with opioid use disorder, only about 25% received MOUD treatment (Dowell et al., 2024), and lower rates of treatment have been reported among historically and systematically underserved groups (Barnett et al., 2023; Dong et al., 2023).

### HEALing Communities Study background

To reduce OODs through increased community-level adoption of EBPs, the National Institutes of Health and the Substance Abuse and Mental Health Services Administration launched the HEALing (Helping to End Addiction Long-term^®^) Communities Study (HCS). HCS was the largest community-engaged, implementation science research study ever conducted in the addiction field. Sixty-seven communities across four states (Kentucky, Massachusetts, New York, and Ohio) participated in HCS, which tested the effectiveness of the Communities That HEAL (CTH) intervention using a community-level, cluster randomized, waitlist-controlled design (Chandler et al., 2020). Forty-three percent of HCS communities were rural, and all participating communities were highly affected by the opioid crisis. The baseline rate of OOD per 100,000 adults was 39.9 in intervention communities and 41.0 in waitlist control communities (Samet et al., 2024).

The CTH intervention consists of three core components: (1) a compendium of EBPs and implementation resources referred to as the Opioid-overdose Reduction Continuum of Care Approach (ORCCA) (Winhusen et al., 2020), (2) community engagement through coalitions to support data-driven adoption and sustainability of EBPs (Sprague Martinez et al., 2020), and (3) communication campaigns to reduce stigma and raise demand for EBPs (Lefebvre et al., 2020). In collaboration with organizations that provide services directly to individuals meant to benefit from the EBPs, referred to as “partner organizations,” HCS coalitions selected and implemented EBP strategies (i.e., specific interventions that fall under the umbrella of OEND and MOUD EBPs) that aligned with their community needs and resources. HCS coalitions were required to select and implement EBP strategies across three sectors (i.e., health care, behavioral health, criminal legal) and in multiple venues, such as emergency departments, first responder stations, jails, and addiction treatment and recovery facilities (HCS Consortium, 2020).

### Research objectives

Chandler et al. (2023) published a description of HCS coalitions’ selected strategies in intervention communities, and this paper examines the implementation and reach of selected strategies in wait-list control communities. Reach, as defined in the RE-AIM/PRISM framework adapted for the HCS, is the number, proportion, and representativeness of individuals who participate in an intervention (Glasgow et al., 2019; Knudsen et al., 2020). Representativeness is a valuable equity marker and can be operationalized in different ways, including similarity and difference between those who were reached by the intervention and the intended population (Glasgow et al., 2019, 2024). The specific research objectives of this paper are to (1) describe the implementation and reach of OEND and MOUD EBPs selected by HCS coalitions in the wait-list control arm while they received the intervention and (2) compare the demographics of individuals reached by the implemented EBPs to the demographics of individuals in these communities.

## Methods

### HCS design

The HEALing Communities Study was a multi-site, parallel-group, cluster-randomized waitlist-controlled trial implemented across 67 rural and urban communities in Kentucky, Massachusetts, New York, and Ohio (HEALing Communities Study Consortium, 2020). HCS communities consisted of counties, cities, towns, or city/town clusters. Thirty-four communities were randomly assigned to receive the CTH intervention from January 1, 2020—June 30, 2022 (intervention arm) and thirty-three communities received the intervention from July 1, 2022—December 31, 2023 (wait-list control arm). The study’s covariate constrained randomization procedure is detailed in a previous publication (HCS Consortium, 2020). As noted in other study publications, HCS was designed to leverage existing administrative data, including data from Medicaid claims, state prescription drug monitoring programs, and emergency medical services, to test the effectiveness of the CTH intervention (HCS Consortium, 2020). However, administrative data had limitations, including data lags and gaps, which necessitated the enhancement of de novo data collection (Volkow et al., 2022). This paper focuses on implementation data collected from the wait-list control communities during the CTH intervention to help supplement administrative data sources. The HCS protocol (Pro00038088) was approved by Advarra Inc., the single Institutional Review Board (sIRB).

### Implementation and reach data collection procedures

Over the course of the study, HCS researchers developed two complementary REDCap instruments to track EBP implementation and reach: the ORCCA Tracker (ORCCAT) and Reach Tracker. For both instruments, HCS staff entered data into site-specific REDCap databases monthly. The site-specific databases were securely transferred to the HCS Data Coordinating Center for quality review and data analysis. ORCCAT fields used in this analysis include EBP strategies and active implementation. EBP strategies are specific interventions that fall under the umbrella of OEND and MOUD EBPs (see Table 1 and the Supplementary Table). The active implementation field records the date services began to be delivered to individuals intended to benefit from the strategy in at least one service delivery location.

Monthly reach reporting began once an EBP strategy reached active implementation. HCS researchers developed common reach measures for EBP strategies, taking into consideration feasibility for data collection across all sites. In some cases, a proxy for individuals receiving services was used as the common reach measure, such as naloxone units distributed to people. See the Supplementary Table for the full list of reach measures by EBP strategy. Given the monthly reporting protocol, reach counts provided by partner organizations may have included duplicates because some individuals may have received OEND and MOUD services in multiple months.

The Reach Tracker included fields for reporting total reach and reach by sex, age group, and race/ethnicity. Due to the breadth of partner organizations engaged in implementing EBPs and varied capacity to collect and report reach data, no standard protocol was developed for partner organizations to collect demographic data from the individuals they provided services to, nor for partner organizations to submit reach data to the research sites. Partner organizations were encouraged to provide complete data; however, reporting was not mandatory.

### Analytic approach

To summarize the reach of OEND and MOUD strategies implemented in HCS wait-list control communities, we conducted two descriptive analyses using data from the ORCCA and Reach Trackers. The first analysis explored the level of reach reporting and overall reach. We analyzed strategy triads—strategy-sector-venue combinations—as described in a previous publication (Chandler et al., 2023) and calculated the number of strategies that reached active implementation before the end of the intervention period. Then, we calculated the percent of actively implemented strategies for which reach data were reported. For each triad, we determined the maximum number of partner organizations reporting reach over each month of implementation and calculated the average monthly reach.

The second descriptive analysis examined representativeness by aggregating triad-level reach for all OEND strategies and calculating demographic distributions for sex (male, female, other, non-response), age (<18 years, 18–34 years, 35–54 years, ≥55 years, non-response), and race/ethnicity (Hispanic, non-Hispanic Black, non-Hispanic White, non-Hispanic other, non-response). We compared the demographic distributions of individuals reached by OEND EBP strategies to those of the community population from the 2020 Bridged-Race Population Estimates (county-level, *n* = 24) or the 2017–2021 American Community Survey 5-Year Averages (sub-county, *n* = 9) (National Center for Health Statistics, 2022; Census Bureau, 2025). We also aggregated reach data for all MOUD EBP strategies. Then, we used 2021 Medicaid claims data to compare the demographic distributions of individuals reached by MOUD strategies to the demographic distributions of adult Medicaid beneficiaries diagnosed with OUD. All analyses were conducted using SAS software, Version 9.4 (2024, SAS Institute Inc., Cary, NC, USA).

## Results

### Implementation and reach

Two hundred and fifty-one OEND EBP strategies and 223 MOUD EBP strategies were implemented by wait-list control communities from July 1, 2022, through December 31, 2023 (Table 1). Reach data were reported for most actively implemented strategies (98.4% of OEND, 99.1% of MOUD). For OEND EBP strategies, aggregated reach data received from 426 partner organizations indicate that overall an average of 8,951 individuals were reached per month, varying across the seven OEND strategy types ranging from 15 to 4,314 individuals reached per month per strategy. For MOUD EBP strategies, aggregated reach data received from 381 partner organizations indicate that overall an average of 7,531 individuals were reached per month, with a range of two to 3,534 individuals reached per month across the 12 MOUD strategy types.

### Representativeness

For OEND EBP strategies, demographic reach data was reported by approximately 69% of partner organizations (Table 2). Compared to the community population, reach to males was slightly underrepresented (44.1% vs 48.4%) while reach to females was slightly overrepresented (54.0% vs 51.6%). Age distributions differ, with the percent of individuals reached by OEND EBP strategies being greater than the community population for individuals aged 18–34 years (39.7% vs 31.2%) and individuals aged 35–54 years (41.3% vs 31.3%). Reach to non-Hispanic Black individuals exceeded community population representation (20.3% vs 14.5%), and reach was comparable to the community population for Hispanic individuals (8.8% vs 8.6%) and individuals of non-Hispanic other races (4.0% vs 4.1%). For strategies with some demographic reach data reported, approximately 17–19% of records documented non-response for individual sex, age, or race/ethnicity, which included ‘missing’ and ‘prefer not to answer.’

For MOUD EBP strategies, demographic reach data was reported by approximately 85% of partner organizations (Table 3). The distributions of sex were similar for individuals reached compared to the community population of Medicaid beneficiaries with OUD (males: 50.1% vs 53.7%; females: 43.7% vs 46.3%). Age distributions showed lower reach of MOUD EBP strategies compared to the community population for individuals aged 18–34 years (28.3% vs 37.8%) but higher reach to individuals aged 35–54 years (58.1% vs 51.8%) and individuals aged 55 years or older (13.5% vs 10.4%). Reach was lower than community representation for non-Hispanic Black individuals (5.5% vs 13.0%) and comparable for Hispanic individuals (5.3% vs 5.5%) and non-Hispanic individuals of other races (1.3% vs 1.4%). For strategies with some demographic reach data reported, between 0.1% and 14.4% of records documented non-response for individual sex, age, or race/ethnicity.

## Discussion

This descriptive analysis highlighted implementation achievements and gaps. HCS wait-list control communities implemented 474 OEND and MOUD EBP strategies, reaching a monthly average of 8,951 and 7,531 individuals, respectively. The demographic distribution of reach for OEND EBP strategies exceeded the proportion of non-Hispanic Black individuals in the community, which is a noteworthy result considering documented racial and ethnic disparities in naloxone access (Khan et al., 2023). This finding aligns with research indicating that community-driven, multi-sector OEND efforts can effectively reach populations disproportionately affected by opioid overdoses (Giglio et al., 2015; Wenger et al., 2022). Our previous study exploring cultural adaptation of EBP strategies in intervention communities highlights the work of some coalitions to diversify partners to better provide community-based OEND, and, anecdotally, research sites applied insights from intervention communities to CTH implementation efforts in wait-list control communities (Gibson et al., 2025). However, available data do not allow for causal analysis.

In contrast to OEND reach, we found that reach of MOUD EBP strategies was low among younger adults and non-Hispanic Black individuals compared to the demographic distribution of Medicaid adult beneficiaries with OUD in the community. These results are consistent with documented inequities in MOUD access (Cruz & Jegede, 2024; Goedel et al., 2020; Guerrero et al., 2022; Hollander et al., 2021). Factors such as workforce shortages, clinical workflow barriers, stigma toward MOUD, regulatory issues, and health-related social needs, which are bolstered by structural inequities, can hinder the implementation and reach of MOUD services (Barnett et al., 2023; Chatterjee et al., 2022; Dong et al., 2023; Hollander et al., 2021). Our results and the literature point to the need for continued investment in multi-level interventions to address these complex and interrelated barriers to medication treatment, including, for example, exploring options to make methadone more accessible through primary care and pharmacies, scaling up efforts to reduce MOUD stigma, and enhancing responsiveness to patients’ treatment preferences (Calcaterra et al., 2019; Jaffe et al., 2024; Suen et al. 2024).

### Limitations

The *de novo* HCS Reach Tracker and data collection effort have limitations that are important to consider when interpreting results. Due to study timeline constraints, we were not able to engage service delivery organizations in the development of the tracker. However, the tracker achieved high reporting rates, indicating its feasibility and usefulness for community-level reach monitoring. To minimize reporting burden and facilitate representativeness analysis, the Reach Tracker collected data on a limited number of demographic variables, so we are unable to assess reach among all groups designated as special populations within the HCS. Also, it was not feasible to collect end user data for all EBP strategies. So, we used reach proxies for some strategies (e.g., the recipient of a naloxone kit as a proxy for the individual naloxone is administered to), which may over or underestimate reach.

There are also limitations to our analytic approach. Because individuals could receive OEND and MOUD services in multiple months, our monthly counts may include duplicates. Therefore, we could not calculate a total number of individuals reached per strategy, but rather had to calculate an average number of individuals reached per month. Additionally, though most partners collected demographic data, individual missing data (approximately 17–19% for OEND and 0–14% for MOUD) due to non-response or selecting the ‘prefer not to answer’ option may affect our results. For the representativeness analysis, all adults with OUD are the intended population for MOUD strategies. However, the best available data source was Medicaid claims, which excludes individuals who are uninsured or have other insurers. Lastly, though HCS was one of the largest and most complex implementation research studies to date, the Reach Tracker was developed as the study evolved and the extent of data lags, gaps and other limitations of planned administrative data sources became clearer (Volkow et al., 2022). Therefore, the tracker was only available for use with the wait-list control communities when they received the intervention later in the project. As a result, we were unable to assess the effectiveness of the CTH intervention on reach.

### Future research

The common reach measures developed for HCS aligned with the best available data that were most feasible to collect across research sites within the study timeline. We share HCS reach measures with the field as a starting point and encourage future formative research to refine a standard set of reach measures for OEND and MOUD EBPs at the community level. Such formative research may involve collaborating with core funders of OUD interventions to explore reach data collection and reporting capacity at different types of community organizations that provide OEND and MOUD services, elucidate the resources required to support reach reporting and analysis, assess data quality and breadth, and test the feasibility of harmonizing reach measures. Innovations in data collection approaches and systems integration could also help advance timely reach tracking for EBPs, including, for example, standardized methods to track naloxone distribution to end users. Innovative solutions are also needed to address data lags and gaps in national and regional administrative data, which are key sources for assessing representativeness, an important equity marker (Glasgow et al., 2024; Volkow et al., 2022). Lastly, rigorous research is needed to test the effectiveness of community-engaged and data-driven intervention in improving reach and representativeness for OEND and MOUD EBPs.

## Conclusion

We set out to describe the implementation and reach, including representativeness, of OEND and MOUD EBPs selected by wait-list control community coalitions. Overall, there was a high level of reach reporting across communities, though completeness of demographic data varied, and representativeness results were mixed. Findings point to both the promise of community-engaged and data-driven interventions to reach diverse groups with OEND EBPs, as well as the challenges of overcoming longstanding barriers to implement MOUD EBPs and reach populations in most need of medication treatment. Our work provides practical examples for using a de novo instrument to gather data on common reach measures across multiple research sites and communities in a timely manner and for applying an equity lens to reach analysis.

## Supplementary Material

Supp 1

Supplemental data for this article can be accessed online at https://doi.org/10.1080/10826084.2025.2549496.

## Figures and Tables

**Table 1.** Actively implemented Overdose Education and Naloxone Distribution (OEND) and Medication for Opioid Use Disorder (MOUD) strategies for *N* = 33 wait-list control communities participating in the HEALing communities Study.

	Number of Strategies implemented^a^, n	Number of Strategies with Available Reach Data^b^, n (%)	Summed Maximum Number of Partners/Organizations Reporting Any Month^c^, n	Summed Average Number of individuals Reached per Month^d^, n

**Total OEND Strategies**	251	247 (98.4%)	426	8,951
**OEND Strategy Type**				
Active OEND for At-Risk individuals	68	68 (100.0%)	115	4,314
Active OEND at High-Risk Venues	79	79 (100.0%)	152	1,446
OEND by Referral	0	NA	NA	NA
OEND Self-Request	65	64 (98.5%)	102	2,073
Naloxone Availability for immediate Use in Overdose Hotspots	27	26 (96.3%)	40	1,040
Capacity for First Responder Administration	11	9 (81.8%)	16	65
Other^e^	1	1 (100.0%)	1	15
**Total MOUD Strategies**	223	221 (99.1%)	381	7,531
**MOUD Strategy Type**				
MOUD Treatment in Primary Care, General Medical and Behavior Health Settings, Specialty Addiction/Substance Use Disorder Treatment Settings, and Recovery Programs	21	20 (95.2%)	31	3,534
MOUD Treatment in Criminal Justice Settings	10	10 (100.0%)	19	168
Access to MOUD through Telemedicine	7	7 (100.0%)	13	131
Interim Buprenorphine or Methadone or Medication Units	2	1 (50.0%)	2	10
Linkage Programs	65	65 (100.0%)	84	399
Bridging MOUD Medications as Linkage Adjunct	13	13 (100.0%)	18	524
Enhancement of Clinical Delivery Approaches That Support Engagement and Retention	36	36 (100.0%)	63	1,091
Use of Virtual Retention Approaches	0	NA	NA	NA
Use of Retention Care Coordinators	13	13 (100.0%)	31	278
Mental Health and Polysubstance Use integration into MOUD Treatment	3	3 (100.0%)	3	29
Reducing Barriers to Housing, Transportation, Childcare, and Accessing Other Community Benefits for People with OUD	52	52 (100.0%)	116	1,364
Other^f^	1	1 (100.0%)	1	2

%: Percentages may not add up to 100 due to rounding.

Data Snapshot: October 23, 2024.

a Actively implemented strategies are defined as those delivering services to individuals meant to benefit from the EBP strategy in at least one service delivery location.

b Percent calculated as the number of implemented strategies with available reach data out of the total number of implemented strategies.

c Number of reporting partners/organizations for a strategy is set equal to the maximum number of partners/organizations reporting data during any single month of implementation.

d The reach of a single strategy is calculated as the average number of individuals reached per month of implementation. The average number of individuals reached is then summed by strategy type. The sum of the counts for strategy type may not equal the total counts due to rounding.

e Other OEND strategies include care coordination to facilitate connections to OEND receipt.

f Other MOUD strategies include care coordination to facilitate connections to MOUD referrals.

**Table 2.** Available reach data for actively implemented Overdose Education and Naloxone Distribution (OEND) strategies versus community demographic compo-sition for *N* = 33 wait-list control communities participating in the HEALing communities study.

Characteristic	Number of OEND Strategies^a^, n(%)	Summed Maximum Number of Partners/Organizations Reporting Any Month^b^, n	Individuals, n (%)	
			Summed Average Number of Individuals Reached per Month^c^	Adult Population^d^

**Total**	247 (98.4%)	426	8,951	3,772,336
**Sex**	168 (66.9%)	296	6,063	3,772,336
Total Non-Missing^e^			5,059 (83.4%)	3,772,336 (100.0%)
Male			2,231 (44.1%)	1,825,776 (48.4%)
Female			2,730 (54.0%)	1,946,560 (51.6%)
Other			98 (1.9%)	NA
Total Missing^e,f^			1,004 (16.6%)	0 (0.0%)
**Age**	166 (66.1%)	294	6,155	3,772,336
Total Non-Missing^e^			4,973 (80.8%)	3,772,336 (100.0%)
<18 Years			55 (1.1%)	NA
18–34 Years			1,975 (39.7%)	1,178,210 (31.2%)
35–54 Years			2,055 (41.3%)	1,180,392 (31.3%)
55+ Years			888 (17.9%)	1,413,734 (37.5%)
Total Missing^e,f^			1,182 (19.2%)	NA
**Race/Ethnicity**	168 (66.9%)	296	6,168	3,772,336
Total Non-Missing^e^			5,023 (81.4%)	3,772,336 (100.0%)
Hispanic			441 (8.8%)	322,654 (8.6%)
Non-Hispanic Black			1,018 (20.3%)	545,357 (14.5%)
Non-Hispanic White			3,362 (66.9%)	2,750,369 (72.9%)
Non-Hispanic Other			201 (4.0%)	153,956 (4.1%)
Non-Hispanic American Indian or Alaskan Native			17	NA
Non-Hispanic Asian			66	NA
Non-Hispanic Native Hawaiian or Other Pacific Islander			4	NA
More Than One Race			114	NA
Total Missing^e,f^			1,145 (18.6%)	0 (0.0%)

%: Percentages may not add up to 100 due to rounding.

Data Snapshot: October 23, 2024.

a Number of actively implemented strategies with available reach data out of all actively implemented strategies from Menu 1: OEND of the Opioid-overdose Reduction continuum of care approach (ORCCA) Tracker.

b Number of reporting partners/organizations for a strategy is set equal to the maximum number of partners/organizations reporting data during any single month of implementation.

c The reach of a single strategy is calculated as the average number of individuals reached per month of implementation. the average number of individuals reached is then summed across strategies by demographics.

d For communities that represent counties (*n* = 24 of 33), population estimates are from 2020 Bridged-Race Population estimates retrieved *via* https://www.cdc.gov/nchs/nvss/bridged_race.htm. For communities that represent units smaller than counties (*n* = 9 of 33), population estimates are from 2017–2021 American community Survey 5-Year Averages retrieved *via* https://data.census.gov/cedsci.

e Percent out of total individuals asked by partner/organization to report data.

f A missing response means the individual either left the field blank or responded as 'Prefer Not to Answer'.

**Table 3.** Available reach data for actively implemented medication for Opioid Use Disorder (MOUD) strategies versus community demographic composition of medicaid beneficiaries with OUD for *N* = 33 wait-list control communities participating in the HEALing communities study.

Characteristic	Number of OEND Strategies^a^, n(%)	Summed Maximum Number of Partners/Organizations Reporting Any Month^b^, n	Individuals, n (%)	
			Summed Average Number of Individuals Reached per Month^c^	Adult Population with OUD^d^

**Total**	221 (99.1%)	381	7,531	62,025
**Sex**	193 (86.5%)	324	7,311	62,025
Total Non-Missing^e^			7,303 (99.9%)	62,025 (100.0%)
Male			3,660 (50.1%)	33,291 (53.7%)
Female			3,192 (43.7%)	28,734 (46.3%)
Other			451 (6.2%)	NA
Total Missing^e,f^			8 (0.1%)	0 (0.0%)
**Age**	194 (87.0%)	323	7,277	62,025
Total Non-Missing^e^			6,536 (89.8%)	62,025 (100.0%)
<18 Years			11 (0.2%)	NA
18–34 Years			1,846 (28.3%)	23,470 (37.8%)
35–54 Years			3,796 (58.1%)	32,109 (51.8%)
55+ Years			883 (13.5%)	6,446 (10.4%)
Total Missing^e,f^			742 (10.2%)	NA
**Race/Ethnicity**	189 (84.8%)	319	7,261	62,025
Total Non-Missing^e^			6,218 (85.6%)	52,663 (84.9%)
Hispanic			331 (5.3%)	2,890 (5.5%)
Non-Hispanic Black			340 (5.5%)	6,840 (13.0%)
Non-Hispanic White			5,470 (88.0%)	42,205 (80.1%)
Non-Hispanic Other			78 (1.3%)	728 (1.4%)
Non-Hispanic American Indian or Alaskan Native			26	NA
Non-Hispanic Asian			13	NA
Non-Hispanic Native Hawaiian or Other Pacific Islander			5	NA
More Than One Race			34	NA
Total Missing^e,f^			1,043 (14.4%)	9,315 (15.0%)

%: Percentages may not add up to 100 due to rounding.

Data Snapshot: October 23, 2024.

a Number of actively implemented strategies with available reach data out of all actively implemented strategies from Menu 2: MOuD of the Opioid-overdose Reduction Continuum of Care Approach (ORCCA) Tracker.

b Number of reporting partners/organizations for a strategy is set equal to the maximum number of partners/organizations reporting data during any single month of implementation.

c The reach of a single strategy is calculated as the average number of individuals reached per month of implementation. The average number of individuals reached is then summed across strategies by demographics.

d Number of individuals with opioid dependence or abuse January 2021 - December 2021 per Medicaid claims data.

e Percent out of total individuals asked by partner/organization to report data.

f A missing response means the individual either left the field blank or responded as 'Prefer Not to Answer'.
