# Supplementary material for: Reach of Community-Selected Strategies to Reduce Opioid-Related Overdose Deaths in the HEALing Communities Study
Source: Subst Use Misuse. Author manuscript; Available in PMC 2025 Sep 27. (PMC12466881; doi:10.1080/10826084.2025.2549496)
Supplement: Supp 1 [file NIHMS2109506-supplement-Supp_1.docx]

Supplementary Table. Opioid-overdose Reduction Continuum of Care Approach (ORCCA) Evidence-based Practice (EBP) Strategies, Description and Reach Measure from the HEALing Communities Study

| **EBP Strategy and Example** | **HCS Reach Measure** |
| --- | --- |
| **OEND** |  |
| - Active OEND for at-risk individuals and their social networks- OEND proactively distributed through peers (i.e., people in the community with lived experience) | Naloxone units (i.e. 2 doses) distributed to individuals |
| - Active OEND at high-risk venues- naloxone kits provided to people upon release from a correctional facility |  |
| - OEND by referral- care providers give prescriptions for naloxone to an individual at high-risk of opioid overdose | Referrals or prescriptions provided to individuals |
| - OEND self-request- pharmacy standing order programs distribute naloxone kits to individuals who request them | Individuals’ requests for naloxone fulfilled |
| - Naloxone availability for immediate use in overdose hotspots- naloxone box installed in public restrooms | Naloxone units added to naloxone boxes |
| - Capacity for first responder administration- naloxone kits distributed to emergency medical technicians | Naloxone units distributed to first responders |
| **MOUD** |  |
| - MOUD treatment in primary care, other general medical and behavioral health settings, and in specialty addiction/ substance abuse disorder treatment settings and recovery programs- implementing MOUD integration or expansion initiatives in these venues | Individuals receiving MOUD |
| - MOUD treatment in criminal justice settings- implementing MOUD integration or expansion initiatives in these jails, prisons and other criminal justice venues |  |
| - Access to MOUD through telemedicine- building healthcare providers capacity to provide telehealth prescriptions of medications for treatment |  |
| - Interim buprenorphine or methadone or medication units- opioid treatment programs (OTP) dispense methadone or buprenorphine to patients on waitlists for up to 120 days without comprehensive ancillary services |  |
| - Linkage Programs- connecting people who need MOUD to care in priority settings, including syringe service programs, emergency departments) | Referrals to MOUD provided to individuals |
| - Bridging MOUD medications as linkage adjunct- induction on medication treatment during incarceration or immediately prior to release | Individuals receiving at least one dose of MOUD at bridging location |
| - Enhancement of clinical delivery approaches that support engagement and retention- case management or peer support | Contact/visit/touchpoint provided to individuals receiving MOUD support services |
| - Use of virtual retention approaches- use of mobile, web, or digital tools to improve MOUD engagement and retention | Virtual contact with peer or provider to enhance retention provided to individuals in MOUD care |
| - Use of retention care coordinators- integrate retention care coordinators into clinic workflow | Contact/visit/touchpoint provided to individuals receiving MOUD support services |
| - Mental health and polysubstance use integration into MOUD treatment- integrate existing mental health services in the community with MOUD providers | Mental health/polysubstance use staff seeing individuals receiving MOUD support services |
| - Reducing barriers to housing, transportation, childcare, and accessing other community benefits for people with OUD- MOUD providers access community benefits to provide transportation services for individuals receiving medication treatment | Assistance/service provided to individuals receiving MOUD support services |
